# Assessment of biomarkers using multiplex assays in aqueous humor of patients with diabetic retinopathy

**DOI:** 10.1186/s12886-017-0572-6

**Published:** 2017-10-02

**Authors:** Hui Chen, Xiongze Zhang, Nanying Liao, Feng Wen

**Affiliations:** State Key Laboratory of Ophthalmology, Zhongshan Ophthalmic Center, Sun Yat-sen University, Guangzhou, 510060 China

**Keywords:** Aqueous humor, Cytokines, Chemokines, Growth factors, Diabetic retinopathy

## Abstract

**Background:**

With the high prevalence of type 2 diabetes, diabetic retinopathy (DR) has become a leading health problem worldwide. The pathogenesis of DR is complex and several vascular, inflammatory, and neuronal mechanisms are involved. The purpose of this study was to assess the levels of immune and inflammatory biomarkers in the aqueous humor of patients with different severities of DR and to analyze the correlations between Interleukin-6 (IL-6) and these biomarkers, and between IL-6 and the severity of the disease.

**Methods:**

Aqueous humor samples were obtained from 51 non-diabetic patients and 151 diabetic patients. Levels of 45 different cytokines, chemokines, and growth factors were measured using a multiplex bead immunoassay.

**Results:**

IL-6, IL-8, Inducible Protein-10 (IP-10), leukemia inhibitory factor (LIF), hepatocyte growth factor (HGF) and vascular endothelial growth factor (VEGF)-A were significantly higher (*p* < 0.05) in the aqueous humor of the DR patients compared to the non-diabetic patients, while the concentrations of IL-1α, IL-4, IL-9, IL-21, IL-23, IL-27, IL-31, RANTES, interferon-α, growth regulated oncogene (GRO), and tumor necrosis factor (TNF)-α were significantly lower (*p* < 0.05) in the DR patients. The IL-6 levels increased as the severity of DR increased. In addition, the IL-6 level positively correlated with the IL-8, HGF and LIF levels, while negatively with the IL-31and GRO levels.

**Conclusions:**

These findings suggest that inflammation and immune response may contribute to the pathogenesis of DR, and these biomarkers may potentially be new therapeutic targets for DR.

## Background

With the high prevalence of type 2 diabetes, diabetic retinopathy (DR) has become a leading public health problem worldwide [1]. The pathogenesis of DR is complex and several vascular, inflammatory, and neuronal mechanisms are involved. Recently, it is known that the disturbance of macrophages, leucocytes, and inflammatory processes may play an important role in the pathogenesis of DR [2]. However, the molecular mechanisms associated with DR are not fully understood. Previous studies show that deregulation of immune responses associated with diabetes can induce increased expression of various mediators resulting in DR development [3]. Thus, analysis of intraocular humors (e.g., vitreous fluids, tear, and aqueous humor) obtained from DR patients has identified some of the mediators (cytokines, chemokines, and other factors) responsible for the pathogenesis of DR. The immune and inflammatory factors, e.g., vascular endothelial growth factor (VEGF), tumor necrosis factor (TNF)-α, interleukin (IL)-1β, IL-6, IL-8, monocyte chemoattractant protein (MCP)-1, cyclooxygenase-2 (COX-2), and pigment epithelium-derived factor (PEDF) have been observed elevated in both sera and vitreous fluids from patients with DR [4]. IL-8, IL-1ra, VEGF et al. are also found to be increased in aqeuous humor of diabetic patients [5].

Despite extensive research, current studies on cytokines in aqueous humor patients with DR are still limited. Many factors play roles in DR and have multiple interactions that affect its pathogenesis. Most studies have worked on aqueous humor cytokines in patients with proliferative diabetic retinopathy (PDR). However, the dynamic changes in the cytokine levels of aqueous humor in patients with different severities of DR should be thoroughly studied because the process by which DM without retinopathy developing into PDR has a chronic course. In this study, we analyzed the levels of 45 immune and inflammatory biomarkers in aqueous samples with DR by using a multiplex bead immunoassay [6]. Furthermore, correlation analysis was performed, including IL-6 vs other cytokines, and laboratory results vs various stages of DR. Our results may have important implications for both development of diagnostic tools and design of potentially new therapeutic targets for treatment of DR.

## Methods

This study was approved by the Ethics Committee of Zhongshan Ophthalmic Centre (ZOC), Guangzhou, China and was performed in accordance with the Declaration of Helsinki for experiments involving human tissues. Informed consent was received from the patients before they enrolled in the study.

### Study population

We recruited 151 patients with type 2 diabetes (78 men and 73 women) and 51 non-diabetic patients (30 men and 21 women) who were undergoing cataract surgery from January 2015 to April 2016. Patients with the following conditions were excluded from this study: previous intraocular surgery, earlier intravitreal therapies, photocoagulation in the preceding three months, uveitis, trauma, vitreous hemorrhage, and retinal detachment. The mean age of the diabetic subjects was 60.1 ± 8.0 years and was 61.1 ± 7.4 years in the control subjects. All of the patients received a complete ophthalmologic examination and a general physical examination. 151 diabetic patients were divided into three groups: 50 with no apparent retinopathy (NDR), 49 with non-proliferative diabetic retinopathy (NPDR), and 52 with proliferative diabetic retinopathy (PDR) based on the Diabetic Retinopathy Disease Severity Scale. The male to female ratio and mean ± SD age was 24/26 and 58.8 ± 8.9 in NDR, 20/29 and 63.2 ± 7.2 in NPDR, and 34/18 and 58.4 ± 7.2 in PDR.

### Sample collection

A limbal paracentesis was performed with a sterile syringe before making the initial incision during cataract surgery. Undiluted aqueous humor samples (100–200 μl) were collected from the paracentesis site under a sterile condition in the operating room. In some patients, sera were collected as well. The samples were immediately transferred to a sterile plastic tube and stored at −80 °C until further study.

### Analysis of cytokines in aqueous humor and serum samples

The cytokines in aqueous humor and sera were measured as described by Chen et al. [7]. The levels of 45 human aqueous humor mediators analysis were measured by a MAGPIX instrument (Luminex Corporation, Texas, USA) and a ProcartaPlex Human Cytokine/Chemokine/ Growth Factor Panel (eBioscience San Diego, CA, USA): (1) the cytokines: IL-1RA, IL-1β, IL-1α, IL-2, IL-4, IL-5, IL-6, IL-7, IL-8/CXCL8, IL-9, IL-10, IL-12 p70, IL-13, IL-15, IL-17A, IL-18, IL-21, IL-22, IL-23, IL-27, IL-31, TNF-α, TNFβ/LTA, interferon-γ (IFN-γ), IFN-α, brain derived neurotrophic factor (BDNF), granulocyte-macrophage colony stimulating factor (GM-CSF), leukemia inhibitor factor (LIF), and stem cell factor (SCF); (2) the chemokines: Eotaxin (CCL11), growth regulated oncogene (GRO)/CXCL1, interferon inducible protein-10 (IP-10)/CXCL10, MCP-1/CCL2, macrophage inflammatory protein-1α (MIP-1α/CCL3), MIP-1β/CCL4, regulated upon activation, normal T cell expressed and presumably secreted (RANTES)/CCL5, and stromal cell derived factor 1α (SDF1α)/CXCL12; (3) the growth factors: epidermal growth factor (EGF), fibroblast growth factor (FGF-2), hepatocyte growth factor (HGF), β-nerve growth factor (β-NGF), platelet derived growth factor (PDGF)-BB, placental growth factor (PLGF), VEGF-A, and VEGF-D. The procedure of analysis was conducted according to the manufacturer’s instructions.

### Statistical analysis

Data are expressed as means ± SD. The data were analyzed using the program SPSS for Windows Version 19.0. The Pearson χ2 test was used to compare the proportions of qualitative variables. Group differences were analyzed between samples from diabetic patients and controls using a student two-tailed t-test or two-tailed Mann–Whitney test based on normality assumptions and homogeneity of variances. Multivariate analysis of covariance (MANCOVA) or the Kruskal–Wallis test was used to compare among multiple groups. Spearman’s rank-order correlation coefficients were used to determine the relationship between the cytokine levels, clinical features, and DR severity. Significant differences were determined at the level of *p* < 0.05.

## Results

### Characteristics of patients with diabetes mellitus

Table 1 shows the demographic and clinical features of the patients, including the NDR and DR patients, and the control group. With regard to age, sex, and body mass index (BMI) distribution, there was no significant difference between the three groups (*p* = 0.291, *p* = 0.5522, *p* = 0.116, respectively). The duration of diabetes was significantly longer in the PDR group than that in both NPDR and NDR groups (*p* < 0.001).

**Table 1:** Clinical and biochemical characteristics of type 2 diabetic patients and non-diabetic control subjects

	Control (*N* = 51)	NDR (*N* = 50)	DR (*N* = 101)	*p*
Sex (m/f)	30/21	24/26	54/47	0.552
Age (years)	61.1 ± 7.4	58.8 ± 8.9	60.7 ± 7.6	0.291
BMI (kg/m^2^)	22.5 ± 2.2	22.6 ± 2.4	23.6 ± 4.7	0.116
Duration (years)	–	8.5 ± 3.8	13.0 ± 3.3	<0.001^*^
FPG (mmol/l)	–	7.5 ± 1.8	9.3 ± 1.8	<0.001^*^
HbAlc (%)	–	8.8 ± 3.5	12.9 ± 3.2	<0.001^*^

Data are expressed as mean ± SD

*DR* type 2 diabetic patients with retinopathy, *NDR* type 2 diabetic patients without retinopathy, *BMI* Body mass index, *FPG* fasting plasma glucose; *HbA1c* glycated hemoglobin

^*^ *p* < 0.05

### Biomarker analysis in aqueous humors

**Fig. 1:**
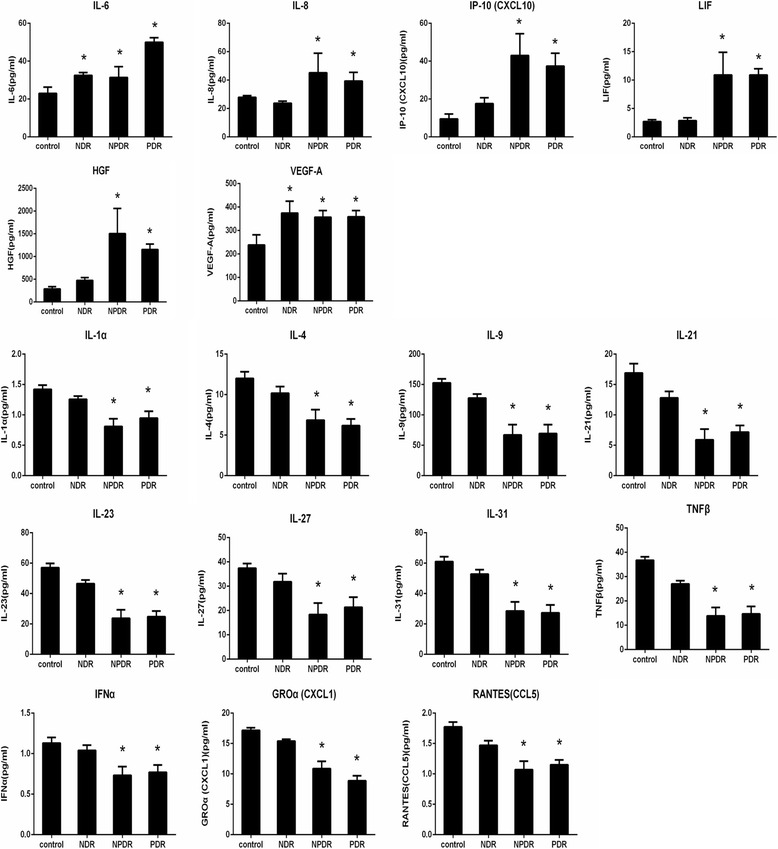
Cytokines levels in the control, NDR, NPDR, and PDR groups. *Statistically significant differences when compared to the control group

The levels of biomarkers, including cytokines, chemokines and growth factors, in each group is summarized in Tables 2, 3 and 4. The DR patients had significantly higher concentrations of IL-6 (*p* < 0.001), IL-8 (*p* < 0.001), IP-10 (*p* < 0.001), HGF (*p* < 0.001), LIF (*p* < 0.001) and VEGF-A (*p* < 0.001) in the aqueous samples compared to the non-diabetic controls and NDR group. However, concentrations of IL-1α (*p* < 0.001), IL-4 (*p* < 0.001), IL-9 (*p* < 0.001), IL-21 (*p* < 0.001), IL-23 (*p* < 0.001), IL-27 (*p* < 0.001), IL-31 (*p* < 0.001) and LIF (*p* < 0.001), RANTES (*p* < 0.001), IFN-α (*p* = 0.001), GRO (*p* < 0.001) and TNF-β (*p* < 0.001) in the DR patients were significantly lower than those in both the control and NDR groups (Fig. 1). IFN-γ was detected in less than 50% of the samples from each group which was not included for further analysis. No significant differences of other cytokines were found between the diabetic patients and the controls. Since diabetic macular edema (DME) was presented in some of the participants, we also compared the difference in the aqueous profile between those with non-DEM. Our results show that these protein factors in aqueous humor significantly increased in the patients with DME (Table 5). Furthermore, we measured the cytokine/chemokine/growth factor levels in sera. Interestingly, all proteins, except VEGF, in sera, significantly increased compared to those in the aqueous humor (Table 6), indicating that the increase is not simply in humor but also systemically.

**Table 2:** Levels of cytokines in aqueous humor

	DR	NDR	Control	*p*
IL-1ra	95.60 ± 93.12	120.71 ± 120.37	88.12 ± 107.15	0.643
IL-1β	1.07 ± 1.03	1.28 ± 1.61	1.86 ± 1.96	0.409
IL-1α	0.88 ± 0.38	1.26 ± 0.24	1.42 ± 0.31	<0.001^*^
IL-2	17.12 ± 13.07	16.66 ± 16.26	17.40 ± 18.35	0.991
IL-4	6.50 ± 3.38	10.17 ± 3.60	11.98 ± 3.77	<0.001^*^
IL-5	1.77 ± 0.98	1.74 ± 1.53	2.45 ± 1.68	0.292
IL-6	40.64 ± 16.52	32.46 ± 6.76	23.00 ± 14.42	<0.001^*^
IL-7	5.00 ± 2.38	4.05 ± 1.46	3.72 ± 1.26	0.143
IL-8	42.20 ± 33.03	23.60 ± 6.84	27.75 ± 5.58	0.011^*^
IL-9	67.92 ± 49.21	127.35 ± 30.46	152.58 ± 28.84	<0.001^*^
IL-10	0.24 ± 0.16	0.16 ± 0.20	0.24 ± 0.28	0.495
IL-12p70	0.30 ± 0.23	0.35 ± 0.51	0.47 ± 0.50	0.621
IL-13	4.46 ± 1.88	5.80 ± 3.38	7.33 ± 3.33	0.083
IL-15	14.42 ± 4.40	14.12 ± 8.20	17.45 ± 6.89	0.298
IL-17A	0.94 ± 0.53	0.98 ± 0.80	1.11 ± 0.82	0.828
IL-18	1.28 ± 0.96	0.81 ± 1.49	1.43 ± 1.71	0.470
IL-21	6.52 ± 4.59	12.77 ± 4.81	16.89 ± 6.91	<0.001^*^
IL-22	183.16 ± 115.29	188.33 ± 133.86	253.94 ± 170.78	0.307
IL-23	24.23 ± 14.56	46.50 ± 10.82	57.02 ± 12.46	<0.001^*^
IL-27	19.82 ± 13.71	31.78 ± 14.88	37.41 ± 8.39	<0.001^*^
IL-31	27.90 ± 17.32	52.73 ± 13.14	60.99 ± 14.53	<0.001^*^
TNFα	4.04 ± 1.83	3.53 ± 1.74	3.77 ± 2.57	0.840
TNFβ/LTA	14.20 ± 10.19	26.94 ± 5.90	36.70 ± 6.44	<0.001^*^
IFNγ	–	–	–	
IFNα	0.75 ± 0.30	1.04 ± 0.29	1.13 ± 0.31	0.001^*^
GM-CSF	25.98 ± 10.31	25.18 ± 13.41	26.75 ± 14.96	0.937
BDNF	1.83 ± 1.24	2.64 ± 0.92	2.81 ± 0.94	0.055
LIF	10.89 ± 9.04	2.86 ± 2.18	2.70 ± 1.35	<0.001^*^
SCF	5.93 ± 3.82	3.72 ± 2.58	4.38 ± 2.74	0.184

Data are expressed as the mean ± SD (pg/ml)

*IL* interleukin, *TNF* tumour necrosis factor, *IFNγ* interferon-γ, *GM-CSF* granulocyte-macrophage colony stimulating factor, *BDNF* brain derived neurotrophic factor, *LIF* leukaemia inhibitor factor, *SCF* stem cell factor

^*^ *p* < 0.05

**Table 3:** Levels of chemokines in aqueous humor

	DR	NDR	Control	*P*
Eotaxin/CCL11	6.61 ± 3.28	5.07 ± 4.40	4.86 ± 3.52	0.510
GROα/CXCL1	9.85 ± 3.33	15.38 ± 1.40	17.17 ± 1.89	<0.001^*^
IP-10/CXCL10	40.12 ± 29.28	17.53 ± 14.03	9.48 ± 11.67	<0.001^*^
MCP-1/CCL2	719.81 ± 598.35	654.68 ± 250.44	593.96 ± 251.10	0.641
MIP-1α/CCL3	2.58 ± 1.99	3.48 ± 2.40	4.66 ± 2.02	0.060
MIP-1β/CCL4	24.18 ± 6.06	29.13 ± 9.50	26.94 ± 4.45	0.233
RANTES/CCL5	1.11 ± 0.35	1.47 ± 0.34	1.77 ± 0.36	<0.001^*^
SDF1α/CXCL12	247.02 ± 54.83	230.00 ± 41.20	212.05 ± 29.15	0.068

Data are expressed as the mean ± SD (pg/ml)

*GRO* growth regulated oncogene, *IP* interferon inducibleprotein, *MCP* monocyte chemoattractant protein, *MIP* macrophage inflammatory protein, *RANTES* regulated upon activation, normal T cell expressed and presumably secreted, *SDF* stromal cell derived factor

^*^ *p* < 0.05

**Table 4:** Levels of growth factors in aqueous humor

	DR	NDR	Control	*P*
EGF	2.10 ± 1.92	1.87 ± 1.37	2.78 ± 1.57	0.193
FGF-2/FGF basic	25.83 ± 12.79	32.36 ± 14.20	25.90 ± 8.51	0.194
HGF	1327.16 ± 1250.12	474.48 ± 267.76	284.67 ± 217.93	<0.001^*^
β-NGF	19.73 ± 14.09	20.51 ± 11.43	25.87 ± 11.90	0.305
PDGF-BB	15.41 ± 14.35	14.44 ± 20.79	18.74 ± 24.66	0.815
PLGF	12.59 ± 2.52	11.50 ± 1.66	12.33 ± 1.94	0.281
VEGF-A	357.02 ± 84.25	373.87 ± 226.73	238.02 ± 192.41	0.039^*^
VEGF-D	5.97 ± 5.20	3.81 ± 3.43	4.88 ± 3.34	0.356

Data are expressed as the mean ± SD (pg/ml)

*EGF* epidermal growth factor, *FGF* fibroblast growth factor, *HGF* hepatocyte growth factor, *NGF* Nerve Growth Factor, *PDGF* platelet derived growth factor, *PLGF* placental growth factor, *VEGF* vascular endothelial growth factor

^*^ *p* < 0.05

**Table 5:** Increased aqueous cytokine concentrations in patients with or without diabetic macular edema

Cytokine	DME (*n* = 55)	Non-DME (*n* = 46)	*P*
IL-6	50.65 ± 6.31	29.11 ± 13.40	<0.001^*^
IL-8	62.04 ± 17.65	36.38 ± 16.76	<0.001^*^
IP-10	54.43 ± 10.83	32.06 ± 11.67	<0.001^*^
LIF	13.81 ± 4.19	6.57 ± 3.46	<0.001^*^
HGF	1513.58 ± 183.90	814.42 ± 326.57	<0.001^*^
VEGF-A	445.40 ± 88.86	326.91 ± 61.29	<0.001^*^

Data are expressed as the mean ± SD (pg/ml)

*DME* diabetic macular edema, *IL* interleukin, *IP* interferon inducibleprotein, *LIF* leukaemia inhibitor factor, *HGF* hepatocyte growth factor, *VEGF* vascular endothelial growth factor

^*^ *p* < 0.05

**Table 6:** Increased cytokine levels in the aqueous humor and serum

Cytokine	Aqueous humor	Serum	*P*
IL-6	33.94 ± 14.40	1.52 ± 0.21	<0.001^*^
IL-8	37.93 ± 20.06	0.66 ± 0.62	<0.001^*^
IP-10	28.52 ± 21.12	9.76 ± 4.12	<0.001^*^
LIF	6.64 ± 5.52	4.06 ± 1.61	<0.001^*^
HGF	786.37 ± 541.07	390.59 ± 100.40	<0.001^*^
VEGF-A	324.65 ± 145.56	393.09 ± 140.59	<0.001^*^

*IL* interleukin, *IP* interferon inducibleprotein, *LIF* leukaemia inhibitor factor, *HGF* hepatocyte growth factor, *VEGF* vascular endothelial growth factor

Data are expressed as the mean ± SD (pg/ml)

^*^ *p* < 0.05

### Correlation analyses in patients with diabetic retinopathy

**Fig. 2:**
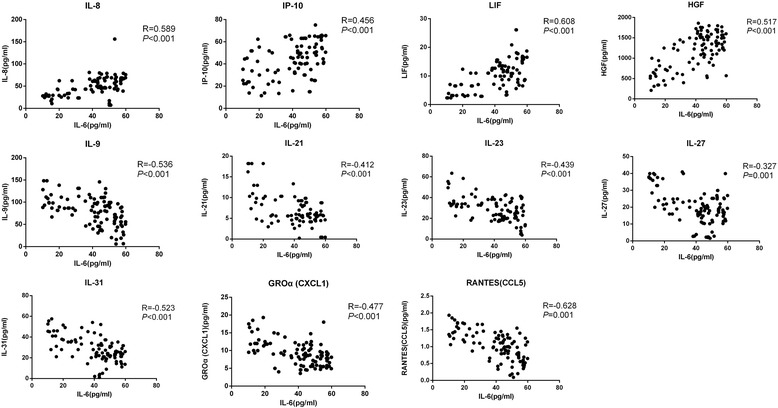
Scatterplot of correlations of IL-6 and other 11 cytokines (IL-8, IP-10, LIF, HGF, IL-9, IL-21, IL-23, IL-27, IL-31, GRO and RANTES) in the DR patients

IL-6 is secreted by T cells and macrophages, which is a multifunctional cytokine and is essential for the regulation of immune response and induction of acute inflammation. It can directly or indirectly contribute to induction of numerous inflammatory cytokines. IL-6 may also causes an increased susceptibility to diabetes mellitus [8]. Therefore, in our study, the correlation of IL-6 with other biomarkers in the aqueous humor was evaluated in the patients with diabetic retinopathy. IL-6 was found to have positive correlations with IL-8, IP-10, LIF, and HGF in the DR group while negative correlations with IL-9, IL-21, IL-23, IL-27, IL-31, GRO and RANTES (Fig. 2). No correlation between IL-6 and VEGF-A was observed (*p* > 0.05). Interestingly, the levels of IL-6 were positively correlated with the DR stage (PDR: 47.68 vs. NPDR: 29.68 pg/ml; *p* < 0.001). Furthermore, the correlations between the duration of diabetes, or HbA1c levels with altered aqueous protein profiles were also analyzed (Tables 7 & 8). In addition, correlation analysis demonstrated that the diabetes duration, HbA1c level, and fasting blood glucose were not associated with any significant alterations in any of the cytokine levels at the time of surgery (*p* > 0.05).

**Table 7:** Correlation between duration of diabetes and altered aqueous cytokines/ chemokines/growth factors in patients with type 2 diabetes

	Cytokines/chemokines/growth factors	Correlation coefficients	*P*
Duration	IL-6	0.350	<0.001^*^
	IL-8	0.563	<0.001^*^
	IP-10	0.444	<0.001^*^
	LIF	0.578	<0.001^*^
	HGF	0.435	<0.001^*^
	VEGF-A	0.324	<0.001^*^
	IL-1α	−0.341	<0.001^*^
	IL-4	−0.383	<0.001^*^
	IL-9	−0.364	<0.001^*^
	IL-21	−0.342	<0.001^*^
	IL-23	−0.324	<0.001^*^
	IL-27	−0.329	<0.001^*^
	IL-31	−0.434	<0.001^*^
	TNF-β	−0.318	<0.001^*^
	IFN-α	−0.203	0.013^*^
	GROα	−0.448	<0.001^*^
	RANTES	−0.296	<0.001^*^

*IL* interleukin, *IP* interferon inducibleprotein, *LIF* leukaemia inhibitor factor, *HGF* hepatocyte growth factor, *VEGF* vascular endothelial growth factor, *TNF* tumour necrosis factor, *IFNγ* interferon-γ, *GRO* growth regulated oncogene, *RANTES* regulated upon activation, normal T cell expressed and presumably secreted

Data are expressed as the mean ± SD (pg/ml)

^*^ *p* < 0.05

**Table 8:** Correlation between HbA1c and altered aqueous cytokines/chemokines/growth factors in patients with type 2 diabetes

	Cytokines/chemokines/growth factors	Correlation coefficients	*P*
HbA1c (%)	IL-6	0.347	<0.001^*^
	IL-8	0.546	<0.001^*^
	IP-10	0.430	<0.001^*^
	LIF	0.567	<0.001^*^
	HGF	0.421	<0.001^*^
	VEGF-A	0.319	<0.001^*^
	IL-1α	−0.341	<0.001^*^
	IL-4	−0.367	<0.001^*^
	IL-9	−0.362	<0.001^*^
	IL-21	−0.339	<0.001^*^
	IL-23	−0.328	<0.001^*^
	IL-27	−0.333	<0.001^*^
	IL-31	−0.432	<0.001^*^
	TNF-β	−0.311	<0.001^*^
	IFN-α	−0.201	<0.001^*^
	GROα	−0.443	<0.001^*^
	RANTES	−0.310	<0.001^*^

*IL* interleukin, *IP* interferon inducibleprotein, *LIF* leukaemia inhibitor factor, *HGF* hepatocyte growth factor, *VEGF* vascular endothelial growth factor, *TNF* tumour necrosis factor, *IFNγ* interferon-γ, *GRO* growth regulated oncogene, *RANTES* regulated upon activation, normal T cell expressed and presumably secreted

Data are expressed as the mean ± SD (pg/ml)

^*^ *p* < 0.05

## Discussion

Previous reports have shown that multiple cytokines and chemokines play crucial roles in DR, suggesting inflammation is a critical factor that is responsible for DR-related changes [6, 9]. Both clinical and laboratory results have demonstrated that DR patients have a high level of inflammatory activity compared to non-diabetic individuals. Analysis of aqueous humor provides useful information for understanding the pathogenesis and treatment responses of various ocular conditions [5]. Though 90 analytes were evaluated recently in diabetic patients, only 2 cases were included in the study [5]. In our study, we compared the levels of 45 cytokines/chemokines /growth factors in aqueous humor of 151 diabetic patients and 51 non-diabetic controls. Also, to our best knowledge, this is the first study in a number of cases to investigate the levels of such a high number of cytokines in the aqueous humor from the patients with DR.

Elevated levels of IL-6 have been reported as a proinflammatory and angiogenic factor in PDR and DM. It has been reported that IL-6 is involved in crossing both the blood brain barrier [10] and the blood-retinal barrier [11, 12]. However, studies of IL-6 levels in the aqueous humor, vitreous, tears, and serum of patients with DR have contradictory results [13]. In our study, the IL-6 levels in the DR patients were significantly increased compared to those in the non-diabetic control group. In addition, the IL-6 level was significantly correlated with PDR. Our results indicate that more serious blood-aqueous barrier breakdown as the proliferative pathogenic process and neovascularization progress. The correlation of IL-6 with other cytokines have also been found (Fig. 2), suggesting that IL-6 may serve as a major driving force that lead to the overall cytokine profile change in the aqueous humor of DR patients. It has been reported that IL-6 can increase vascular permeability and angiogenesis by inducing the expression of VEGF [14]. However, no correlation between the levels of IL-6 and VEGF levels was demonstrated in our study. In previous studies [15], contradictory results have been reported regarding the correlation between IL-6 and VEGF levels in diabetic patients. Our data suggest that IL-6 may not directly link to VEGF in DR. This difference between the two studies could be due to the differences in the samples used (aqueous humor vs vitreous fluid), the assays used (multiplex vs ELISA), or the patients participated (all diabetic patients vs patients with DME), etc. LIF has been shown to play a physiological role in blastocyst implantation and in inflammation [16]. It is reported that LIF modulates VEGF expression and is essential for ensuring proper capillary density [17]. To our best knowledge, our study was the first to detect elevated LIF in aqueous humor of DR.

Chemokines are small heparin-binding proteins that lead to the migration of responding cells [18, 19]. Chemokines are classified into four groups: C, CC, CXC, and CX3C. IL-8, IP-10 and GRO are considered as CXC chemokines. IL-8 is a major chemoattractant that mediate chronic leukocytic inflammation in the vascular walls and eventually leads to capillary occlusion and retinal ischemia [18]. IP-10 is secreted by monocytes, endothelial cells, and fibroblasts, which enhanced the T-helper type 1 immune reactivity. In accordance with previous reports, the IL-8 and IP-10 levels in aqueous humors from the DR patients were significantly increased compared to those of NDR and control groups [18, 20]. It has been shown that levels of vitreous IP-10 were positively correlated with increased VEGF levels [21], however, no association between VEGF and IP-10 in the aqueous humor has been found in our study. CXCL-1/GRO is a chemokine that attracts neutrophils and induce the action of inflammation and angiogenesis [22]. Increased GRO level in the plasma and vitreous of diabetic patients with PDR have been reported in previous study [23]. We consider that GRO may also be an influence factor in DR based on these reports and the result of our study. RANTES is known to be a potent angiogenic factor and induced retinal neovascularization in diabetic patients. In our studies, RANTES level of the DR patients was significantly higher compared to that of controls [24]. Our findings are supported by other studies detected vitreous sample from DR patients [6, 25].

VEGF is one of the key cytokines that induces vascular permeability and angiogenesis, causing the pathogenesis of PDR and diabetic macular edema [26–28]. Also, the VEGF level in vitreous is significantly correlated with DR severity [29]. Our results showed that VEGF level was significantly elevated in diabetic subjects compared to non-diabetic control group. However, VEGF was not correlated with the severity of DR (*p* = 0.357). One explanation for this difference is that severe PDR patients were relatively rare in our study. In addition, VEGF level in the aqueous humor may not absolutely parallel to its level in the vitreous.

HGF is a cytokine that exhibits multiple functions, e.g., regulates cell growth, cell motility, and morphogenesis of various types of cells [30, 31]. High concentrations of HGF in vitreous of patients with PDR have been found [32, 33]. A correlation between HGF levels in the aqueous humor and the presence of fibrovascular membranes in PDR patients was also observed. Consistent with the previous study with vitreous humor samples [33], no correlation was found between the levels of HGF and VEGF in aqueous humor in our study. Our findings suggest that HGF-related fibroproliferative processes or wound healing may be involved in the development of DR.

Our results show that the decreased levels of IL-1α, IL-4, IL-9, IL-21, IL-23, IL-27, IL-31, and IFN-α in the diabetic group (Table 2), while their functional implications still remain unknown. These cytokines are major immunity cytokines that mediate the adaptive immune response. The impairment of the immune balance is likely to induce some of the pathological changes in DR patients. We also observed that decreased levels of TNF-β in aqueous humor of diabetic subjects, while other studies have observed TNF-β were higher in plasma of the diabetic group [34, 35]. We believe that TNF-β may act as a protective factor against retinal damage in DR. The specific roles of TNF-β and other decreased cytokines need to be further investigated to understand the relationship between the decreased level of the protein factors and development of diabetic retinopathy.

## Conclusions

In summary, we have demonstrated significant alteration of immune and inflammatory biomarkers in aqueous humor from diabetic patients with retinopathy. In addition, these biomarkers are found to have significant correlations with IL-6, which was associated with the severity of DR. However, we do not have direct evidence in our study and, therefore, cannot conclude that IL-6 is in any way causative of retinopathy progress. Our results support the hypotheses that chronic inflammation and disturbance of immune system plays an important role in the pathogenesis of diabetic retinopathy. These results may have important implications for design of potential therapeutic targets for DR.

## Abbreviations

BDNF: brain derived neurotrophic factor
CCL11: eotaxin
COX-2: cyclooxygenase-2
DR: diabetic retinopathy
EGF: epidermal growth factor
FGF: fibroblast growth factor
GM-CSF: granulocyte-macrophage colony stimulating factor
GRO: growth regulated oncogene
HGF: hepatocyte growth factor
IFN-γ: interferon-γ
IL: interleukin
IP-10: interferon inducible protein-10
LIF: leukemia inhibitor factor
MCP: monocyte chemoattractant protein
MIP-1α: macrophage inflammatory protein-1α
NDR: no apparent retinopathy
NPDR: non-proliferative diabetic retinopathy
PDGF: platelet derived growth factor
PDR: proliferative diabetic retinopathy
PEDF: pigment epithelium-derived factor
PLGF: placental growth factor
RANTES/CCL5: regulated upon activation, normal T cell expressed and presumably secreted CCL5
SCF: stem cell factor
SDF1α: stromal cell derived factor 1α
TNF: tumor necrosis factor
VEGF: vascular endothelial growth factor
β-NGF: β-nerve growth factor
